# The Impending War

**Published:** 1861-07

**Authors:** 


THE IMPENDING WAR.—America is upon the verge of a mighty revolution. The fiat of war has gone forth; the fife and the drum are heard in our midst; armed men are rushing to and fro; nearly fifty thousand soldiers are guarding the capital of the Union; a large opposing military force is marching from the South; and it requires no special sagacity to perceive that a terrible conflict is about to be inaugurated—a conflict destined, in all likelihood, to be more sanguinary than any since the days of the first Napoleon. Our country is placed under new and startling relations. For nearly half a century it has enjoyed uninterrupted peace, and an amount of prosperity unequaled by that of any other nation of the globe. The art of war had literally slumbered with the heroes of the Revolution. The sword and the musket had been exchanged for the plowshare and the pruning-knife. Agriculture, the mechanic arts, and the sciences possessed unrivaled sway. We had, it is true, during this period, some hard Indian fights, and we were compelled, or thought ourselves compelled, to send an army into Mexico, in order to put an end to her hostile incursions upon our Southern frontiers. But these were mere collateral issues, scarcely in any degree affecting the national weal. Now, however, the American people are about to be plunged into civil war; a war which God alone, in his wisdom and mercy, can avert, and of which, if it be once fully inaugurated, it is impossible to foresee the end, however easy it may be to perceive the dreadful consequences both to ourselves and to civil liberty throughout the world.

Amid these scenes of strife and distraction, science shrinks from her accustomed walks, and seeks the retirement of the closet and the cloister. Her pupils are scattered far and wide; the halls of the academy are forsaken; and the press, her willing instrument in times of peace, no longer requires her aid. Her voice is silent, and her disciples go mourning about the street. The interests of our own profession are deeply involved in the sad consequences of the impending war. Already a number of journals have been suspended, either on account of the disturbed state of the postal arrangements, or because the editors have laid down the pen for the sword; and it is easy to foresee that our medical schools, both North and South, will have diminished classes during the next session. A great demand has, it is true, been created for military and naval surgeons, but by far the greater proportion of our students will enter the service of the country in the capacity of privates, preferring to fight in the ranks to waiting for the tardy acquisition of the medical diploma.

The approaching contest will undoubtedly afford, what it is too sad to contemplate, ample opportunities to our surgeons for the exercise of their skill, humanity, and bravery. War is well calculated to develop the noblest traits of their character. Their duties are immense; for not only are they to take care that the men who are committed to their charge do not become sick from the use of improper food, bad clothing, and unnecessary exposure and fatigue, but should any fighting occur they must be prepared, with all the readiness and alacrity of experts, to do justice to the wounded, affording them all the aid and comfort of the art and science of their sacred calling. The qualifications of men destined for the army and naval service of the country cannot be too closely scrutinized; none should be admitted who are not thoroughly competent for every emergency, however sudden, unexpected, or trying. Disease, if not the sword, will, in any event, during the present crisis, slay its thousands; and let the authorities see that the medical men whom they send to take care of our troops do not add, by their want of knowledge and courage, to the horrors of the mortality.

America has never produced one great military surgeon, or one who has made, from the storehouse of his own experience, any important contributions to our stock of knowledge. One reason of this, no doubt, is that we have not been engaged in any great battles such as those which have so often desolated the nations of Europe. Under more favorable circumstances for distinction, there is no reason to believe that our surgeons, army as well as naval, would not, like those of the Old World, have covered themselves with glory. The British army has been fruitful in great surgeons. The names of Woodall, Wiseman, Brown, Hunter, Hennen, Thomson, S. Cooper, Guthrie, McGrigor, Ballingall, Hutchinson, Alcock, Burnett, and Macleod, not to mention many others, have shed imperishable lustre upon British medicine.

